# Venomics of Scorpion *Ananteris platnicki* (Lourenço, 1993), a New World Buthid That Inhabits Costa Rica and Panama

**DOI:** 10.3390/toxins16080327

**Published:** 2024-07-23

**Authors:** Cecilia Díaz, Bruno Lomonte, Arturo Chang-Castillo, Fabián Bonilla, Adriana Alfaro-Chinchilla, Felipe Triana, Diego Angulo, Julián Fernández, Mahmood Sasa

**Affiliations:** 1 Instituto Clodomiro Picado, Facultad de Microbiología, Universidad de Costa Rica, San José 11501-2060, Costa Rica; bruno.lomonte@ucr.ac.cr (B.L.); arturo.chang@ucr.ac.cr (A.C.-C.); fabian.bonilla@ucr.ac.cr (F.B.); adriana.alfaro15@ucr.ac.cr (A.A.-C.); andres.triana@ucr.ac.cr (F.T.); luis.angulocastro@ucr.ac.cr (D.A.); julian.fernandezulate@ucr.ac.cr (J.F.); mahmood.sasa@ucr.ac.cr (M.S.); 2 Departamento de Bioquímica, Escuela de Medicina, Universidad de Costa Rica, San José 11501-2060, Costa Rica; 3 Museo de Zoología, Centro de investigación de Biodiversidad y Ecología Tropical, Universidad de Costa Rica, San José 11501-2060, Costa Rica

**Keywords:** *Ananteris*, scorpion, venomics, Buthidae, toxin

## Abstract

*Ananteris* is a scorpion genus that inhabits dry and seasonal areas of South and Central America. It is located in a distinctive morpho-group of Buthids, the ‘*Ananteris* group’, which also includes species distributed in the Old World. Because of the lack of information on venom composition, the study of *Ananteris* species could have biological and medical relevance. We conducted a venomics analysis of *Ananteris platnicki*, a tiny scorpion that inhabits Panama and Costa Rica, which shows the presence of putative toxins targeting ion channels, as well as proteins with similarity to hyaluronidases, proteinases, phospholipases A_2_, members of the CAP-domain family, and hemocyanins, among others. Venom proteolytic and hyaluronidase activities were corroborated. The determination of the primary sequences carried out by mass spectrometry evidences that several peptides are similar to the toxins present in venoms from Old World scorpion genera such as *Mesobuthus*,* Lychas*, and *Isometrus*, but others present in *Tityus* and *Centruroides* toxins. Even when this venom displays the characteristic protein families found in all Buthids, with a predominance of putative Na^+^-channel toxins and proteinases, some identified partial sequences are not common in venoms of the New World species, suggesting its differentiation into a distinctive group separated from other Buthids.

## 1. Introduction

Scorpions are an ancient clade of arachnids, taxonomically divided into 24 extant families. Buthidae is the largest in number of species, and the most studied due to the medical importance of its venoms [1,2]. Within Buthidae, at least six groups have traditionally been recognized: *Ananteris*, *Buthus*, *Charmus*, *Isometrus*, *Uroplectes*, and *Tityus*, based on external morphological characters [3]. While the *Buthus* and *Tityus* groups constitute well-supported clades in most phylogenetic reconstructions, the others present a fascinating challenge as they exhibit inconsistencies in their phylogenetic relationships that suggest they may not be natural groupings [4,5].

The *Ananteris* group was first established to include several species of buthid scorpions with the following combination: absence of fulcra in their pectins, telson fusiform in shape, orthobothriotaxic trichobothria pattern, fingers of the chela with 6–7 rows of granules, and weak sexual dimorphism [6,7,8]. Traditionally, this group has been made up of small species distributed in tropical America (genera *Ananteris* and *Microananteris*) and others with distribution in Africa and Asia (genera *Ananteroides*, *Tityobuthus*, *Lychas*, *Lychasioides*, *Himalayotityobuthus*, and *Troglotityobuthus*). Recently, Ythier [9] proposed that the distinction of these genera was so remarkable that it would allow the group to be recognized as a valid family, Ananteridae Pcocock, 1900, separated from Buthidae. However, the relationships among the presumed members of this new family and with other scorpions recognized as buthids are still not entirely clear, so we refrain from following this possibility until more information is available.

Botero-Trujillo and Noriega [10] synonymized the monotypic genus *Microananteris* with *Ananteris,* Thorell, 1891, so the latter is the only genus of the group currently recognized in the New World.

The distribution of existing and fossil species in the *Ananteris* group has led to the notion that it corresponds to an ancient lineage with Pangean distribution patterns [8]. Affinities between *Ananteris* and *Lychas* have been proposed based on morphological [8] and molecular evidence [11]. Furthermore, *Lychas* has been phylogenetically associated with another genus of Old World Butidae, *Isometrus* [4,12], a scorpion of Asian origin with a species (*I. maculatus*) that has dispersed all over the world through human commercial activities [13,14].

Recently, Stundlová and collaborators [4] have questioned the monophyly of the *Ananteris* group, showing that *Ananteris* and *Lychas* have affinities with members of the *Isometrus* group. This notion, however, does not preclude the close evolutionary relationship between the genera included within the *Ananteris* group. Moreover, it has been suggested that members of this group, and particularly the genus *Ananteris*, share a common ancestor with the other buthids distributed in the New World, such as the diverse genus *Tityus*, owing to its wide distribution and the probability that the ancestor existed before the separation of the continents [15]. In support of this view, most identified amber fossils, all of which belong to the family Buthidae, are closely related to extant members of the *Ananteris* group, which is widely considered a basal lineage [16]. Therefore, despite its Neotropical distribution, the genus *Ananteris* appears to have an Old World origin [8].

Currently, *Ananteris* includes approximately 96 species [9] that inhabit dry, seasonal, and even humid regions of the Neotropics, from Costa Rica to Paraguay. The genus was initially created to describe *A. balzanii,* Thorell, 1891, from Brazil. Shortly after, two new species were incorporated, *A. cussinii*, Borelli, 1910, and *A. festae*, Borelli, 1899. As Ythier [9] points out, the number of species of this genus increased considerably in the last four decades, with the highest species richness found in Venezuela [17], Colombia [18], and Brazil [19]. Members of this genus have relatively small bodies (<30 mm) and maintain notable anatomical similarities.

Despite their remarkable diversity, there are no reports on venom composition for any species of *Ananteris*, probably due to the small size and secretive habits in the leaflitter characteristic of most members of the genus. This lack of studies prevents any light from being shed on the potential medical importance and possible toxinological relationships of *Ananteris* species with other buthid scorpions. In contrast, transcriptomic analyses of the venom gland of the related scorpion *Lychas mucronatus* have been performed [20,21]. Venom-associated components for this species include several peptides, such as lipolysis-activating peptides, Na^+^ and K^+^ channel-modulating peptides, calcins, La1-like peptides, protease inhibitors, host defense peptides, and, as in the case of all known buthid venoms, enzymes such as metalloproteinases, serine proteinases, and phospholipases A_2_, are also present [20,22]. Some other proteins are found in *L. mucronatus*; among them, the characteristic SCP domain-containing proteins are present, such as CAP-Lyc1 [20], which belong to the same family of CRISP and insect allergens [23,24]. Some previously identified and characterized *L. mucronatus* ion channel-modulating toxins show specificity toward insect targets [25], but others could potentially affect mammalian channels [26,27].

Due to their phylogenetic proximity, a similar venom composition could be expected for species of the genus *Ananteris*. Analyzing the venom composition of members of this genus could not only elucidate the relevant aspects of their potential toxicity to humans but also facilitate the understanding of their evolutionary diversification to other lineages of buthid scorpions, both from the Old and the New World.

As part of an ongoing effort to understand venoms from Central American scorpions, we performed a proteomic analysis of *Ananteris platnicki,* Laurenco, 1993, a small species distributed in the humid and premontane forests of Costa Rica and Panama. *A. platnicki* is an inhabitant of the forest floor, where it is found associated with fine leaf litter and rotting logs in secondary growths. This species follows a clustered distribution throughout its geographic range, making it abundant in some localities. However, little is known about its ecology and behavior [28]. Our venomics study shows that *A. platnicki* expresses the majority of the characteristic toxin families found in the New World Buthidae genera. It reflects closer proximity to the venom of the *Lychas* and *Isometrus* species, reinforcing the currently accepted view for the phylogenetic position of the genus *Ananteris*.

## 2. Results

**Figure 1:**
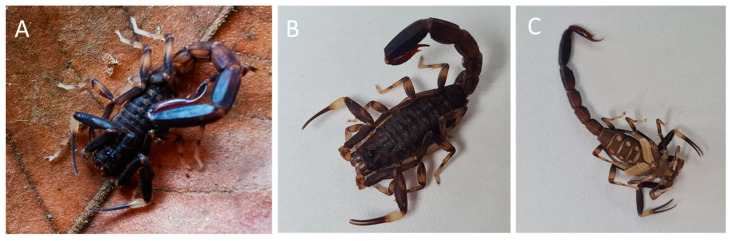
(**A**) *Ananteris platnicki* specimen in the field in Hacienda Barú, in Puntarenas Province of Costa Rica; (**B**) frontal picture of collected *A. platnicki* specimen; (**C**) dorsal picture of collected *A. platnicki* specimen.

*Ananteris platnicki* scorpions were observed active at night in the leaf litter of a recovering secondary forest. All specimens were collected in an area of approximately 600 m^2^. The identification of the specimens followed the description of Lourenço [29], which emphasizes the elongated telson and the characteristic brown body coloration (including pedipalps) with legs, hands, and chelicerae of a light-yellow color (Figure 1). Pectins without fulcra were observed in the ventral image, which is characteristic of ‘*Ananteris*-group’ members [30] (Figure 1C).

**Figure 2:**
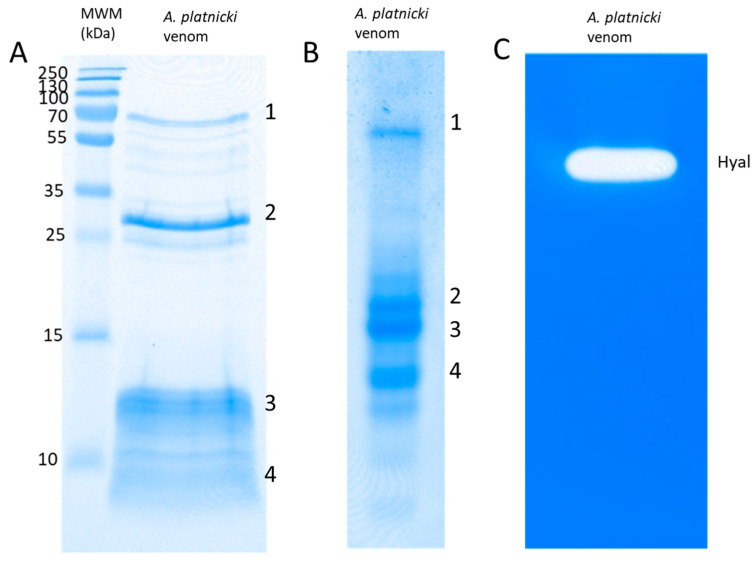
(**A**) Protein pattern of a venom pool from *Ananteris platnicki* specimens examined by SDS-PAGE (reducing conditions); (**B**) PAGE under native conditions (without SDS or β-mercaptoethanol); (**C**) hyaluronidase activity by zymography. The numbers on the right side in (**A**,**B**) represent MS-analyzed bands for identification of specific components, whose presence was confirmed in the ‘shotgun’ analysis of the crude venom (see the Results Section).

During venom extraction, three individuals autotomized their abdomen between the second and third metasoma segments, a behavior that has been reported for members of this genus [31]. Extracted venom consists of a thick, whitish liquid. In the venom SDS-PAGE (Figure 2A), most of the peptides are located below 15 kDa, and the higher molecular weight compounds include two distinctive bands at around 75 and 30 kDa. The general composition of these two leading bands is represented (at least partially) by venom hemocyanins and their fragments, respectively (see below). Under native conditions, the venom separates into at least four distinctive bands (Figure 2B), with a general composition described below.

Mass spectrometry analysis of the main venom components grouped by families is presented in Table 1 and described in more detail in Table S1. The classification of the families is similar to other buthid venoms, with putative ion channel-modulating toxins (Na^+^ and K^+^, mainly) and several lipolysis-activating peptides (LAP, α- and β-subunits). In the case of the putative K^+^ channel-modulating compounds, we identified a peptide (partial sequence LNKKCNSDSDCCR) very similar to a toxin present in *Mesobuthus eupeus* venom and other Old World scorpions, which has also been associated with the Ca^+2^-channel activation of nuclear inositol 1,4,5-triphosphate receptors in cardiomyocytes [32].

**Figure 3:**
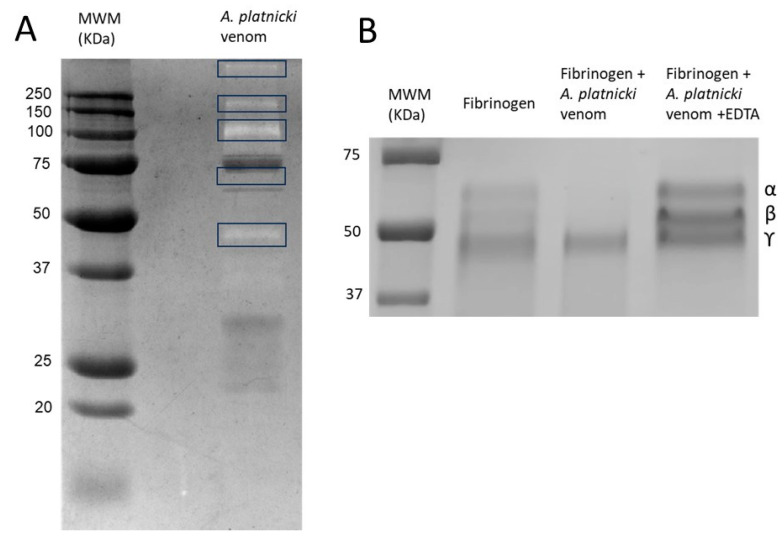
(**A**) Proteolytic activity of *Ananteris platnicki* venom on gelatin examined by zymography; and (**B**) fibrinogenolytic activity on human fibrinogen examined by SDS-PAGE. In (**A**), the main gelatinolytic enzymes are framed for better observation. The experiment presented in (**B**) shows the disappearance of fibrinogen α- and β- chains as the result of incubation with *A. platnicki* venom, without affecting the ϒ-chain. Also, it is observed the inhibitory effect of the metalloproteinase inhibitor EDTA on the fibrinogenolytic activity of the venom.

**Figure 4:**
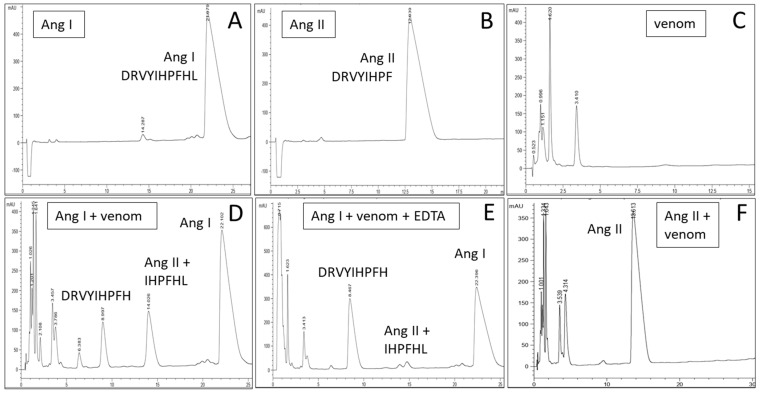
Angiotensin-converting enzyme activity of *Ananteris platnicki* venom determined by RP-HPLC. (**A**) Human angiotensin I was used as a substrate. (**B**) Human angiotensin II was used as a standard. (**C**) *A. platnicki* venom. (**D**) *A. platnicki* venom incubation with angiotensin I, showing the formation of several peptides, including angiotensin II. (**E**) *A. platnicki* venom incubation with angiotensin I in the presence of metalloproteinase inhibitor EDTA, showing practically no angiotensin II formation, only the presence of a peptide probably resulting from the activity of other venom proteases on angiotensin I. (**F**) *A. platnicki* venom incubation with angiotensin II, showing no signi-icant proteolytic activity of the venom. Peptides derived from angiotensin I treated with the venom were identified and confirmed by MS analysis (see the text).

Some enzymatic activities of *A. platnicki* venom were confirmed. It displays hyaluronidase (Figure 2C) and proteolytic activities (Figure 3), including the ability to process α- and β-fibrinogen chains (Figure 3B). Regarding proteolytic activity, we observed the ability of the venom to cleave human angiotensin I into angiotensin II (Figure 4). In that experiment, other peptides were formed that were detected by HPLC (Figure 4D) and that could correspond to other fragments cleaved from substrate angiotensin I and/or its products by other proteases present in the venom, except for angiotensin II, which we confirmed remained intact after incubation with the venom (Figure 4F). EDTA inhibited venom-induced angiotensin II, presumably by chelating cofactor ions for the ACE-like enzyme (Figure 4E). EDTA also inhibited the formation of a newly identified peptide, IHPFHL, but not the removal of the C-terminal leucine from angiotensin I, since the peptide DRVYIHPFH produced by the proteolytic activity of the venom remained intact in the presence of the inhibitor (Figure 4D,E).

**Figure 5:**
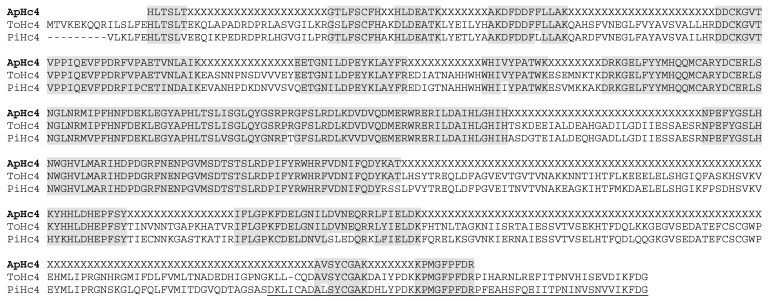
Alignment of the amino acid sequences of *Ananteris platnicki* hemocyanin 4 compared to the same subunit sequences reported for *Tityus obscurus* and *Pandinus imperator*. Strictly conserved amino acids are shaded in gray. ApHc4: *A. platnicki* subunit 4; ToHc4: *T. obscurus* subunit 4; PiHc4: *P. imperator* subunit 4. About 50% of the *A. platnicki* venom hemocyanin 4 sequence was identified by MS. The underlined sequence at the C-terminal of *P. imperator* displays 97% similarity to a transcript identified in *Pandinus cavimanus* venom gland, demonstrating that hemocyanin fragments are not necessarily contaminants, but are potentially expressed in scorpion venoms.

In the venom, we also identified several putative enzymes such as phospholipases A_2_, amylases, glyceraldehyde-3-phosphate dehydrogenase, phosphodiesterase, and peptidylglycine-α-hydroxylating monooxygenases. We found members of the venom ubiquitous CAP-domain family such as the cysteine-rich proteins, some secreted venom proteins of unknown function in scorpion venoms, and several hemocyanin subunits (Table 1). Among the latter, we identified peptide sequences from the eight highly conserved scorpion subunits, classified as 2, 3a, 3b, 3c, 4, 5a, 5b, and 6, with homology to several scorpion species [33]. Figure 5 presents the peptide sequences obtained from *A. platnicki* hemocyanin 4, aligned together with the *Tityus obscurus* and *Pandinus imperator* sequences obtained from public databases.

**Figure 6:**
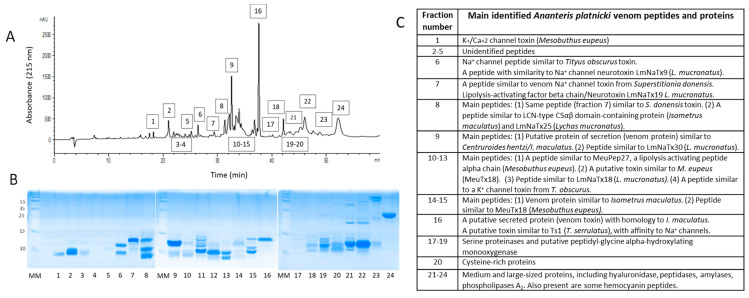
(**A**) RP-HPLC separation of *Ananteris platnicki* venom applying a linear acetonitrile gradient during 60 min. (**B**) RP-HPLC peptide and protein-containing fractions obtained from the venom analyzed by SDS-PAGE under reducing conditions. MM: Molecular markers. (**C**) A table containing the main identified *A. platnicki* venom peptides and proteins found in RP-HPLC fractions and obtained from MS analysis of SDS-PAGE bands (see Table 1). Each number in Figure 6C corresponds to the numbers from the bands at the lower part of the gel in 6B, and the respective fraction numbers at the 6A RP-HPLC chromatogram. All the identified components are included also in Table 1.

The venom was separated by RP-HPLC and the main fractions were further analyzed by SDS-PAGE to be individually sequenced and assigned to protein families by comparison (Figure 6). We corroborated the presence of most of these components from the initial venom ‘shotgun’ analysis (Table 1). Not all the components were identified, but most of them corresponded to the LAPs, secreted venom proteins, and putative ion channel-modulating peptides. The first eluted RP-HPLC peptide was similar to the *M. eupeus* K^+^/Ca^+2^ toxin, and the last band corresponded mainly to hemocyanins, which, according to the SDS-PAGE molecular weight, probably represent only fragments [34].

Most of the identified peptides (Table 1 and Figure 6C) show similarity to *Lychas mucronatus* and *Isometrus maculatus* venom compounds. Some proteins show similarities to enzymes from other scorpion species, such as *Hadrurus spadix* and *Centruroides *spp.

Our sequence analysis extended to the venom bands from the SDS-PAGE and the native-PAGE (Figure 2). As previously mentioned, the SDS-PAGE (Figure 2A) revealed a conspicuous band (band 1, ~75 Kda) that contains multiple hemocyanin subunits, congruent with the expected masses from other scorpion species (Table S1). Additionally, SDS-PAGE band 1 harbored putative proteolytic enzymes with an expected mass between ~70 and 80 KDa.

According to Figure 3A, the gelatinolytic activity was only associated with the lower part of this band (Figure 2A), indicating that these proteases, including the putative angiotensin-converting enzyme (ACE-like), may not constitute a significant portion of the band.

The other SDS-PAGE prominent band (band 2, ~30 kDa) contains also several hemocyanins (possibly fragments) and proteins from the CAP-domain family, secreted PLA_2_s, and metalloproteinases, all present in Table 1. According to their putative homologs in other scorpion venoms, these proteins could have masses between 25 and 47 kDa (Table S1). No proteolytic activity on gelatin corresponded to this band (Figure 2A and Figure 3A).

The other SDS-PAGE bands, which include smaller-size components, contain the putative LAPs and Na^+^-channel-modulating toxins and the putative K^+^ channel toxins (Figure 2A), which correspond with the expected masses for these peptides in other scorpion venoms (Table S1).

When analyzing the bands from native-PAGE (Figure 2B), one of the findings of its composition was the presence of CAP-Lyc-1 and hyaluronidase migrating together in band 1. This is interesting because it has been suggested that in the venom of some arthropods, these kinds of proteins could form a complex [35]. This gel band also contains two putative LAPs, an α-chain and a β-chain, which could suggest that both peptides could be subunits of the same protein. Native-PAGE bands 2 and 3 contain mainly putative peptidase S1, peptidase M14 (see Table 1), and secreted venom proteins with functions still unassigned (venom proteins from Table 1 and Table S1). In band 4, we identified another putative metalloproteinase. The four fractions from the native-PAGE also contained hemocyanin subunits or their fragments (Figure 2B).

## 3. Discussion

Buthid venoms display a characteristic general composition (protein families) with an abundance of ion-modulating peptides, especially Na^+^ channel toxins [22], and different types of proteolytic enzymes [36,37]. Their venoms also include other proteins, with and without enzymatic activities, protease inhibitors, and antimicrobial peptides, since biological functions are related to predation and defense against predators and pathogens [38].

The protein and peptide venom profile of *A. platnicki* is likely tailored to arthropod targets, mainly through channel-modulating Na^+^ and K^+^ toxins, which can paralyze and potentially kill insects [39] but also possible predators [40]. The specificity of these toxins, as demonstrated by recombinant toxins from the phylogenetically close species *Lychas mucronatus* [25], emphasizes this adaptation. However, it is worth noting that peptides targeting vertebrate ion channels could also be present [26,27], potentially serving as a defense mechanism [41].

Within the group of Na^+^ channel toxins, other components in the venom of *A. platnicki* were the lipolysis-activating peptides (LAP), with at least three putative variants, two α- and one β-subunit. These peptides are commonly found in Old World scorpions [42]. In contrast, only α-subunits have been discovered in New World buthids, mainly in the transcriptome of some *Tityus* and *Centruroides* species [43,44]. Also, it has been suggested that a 7-Cys LAP α-subunit was probably the ancestor protein of the Na^+^ channel-modulating toxins [45].

LAPs found in *A. platnicki* have similarities to *Lychas mucronatus* LmNaTx25 (LVP2-α) and LmNaTx19 (LVP1-β) chains, which could probably be subunits of the same heterodimeric protein. Another putative toxin was also identified, with an identity to MeLVP1-α from *Mesobuthus eupeus* (Meupep27) [42]. Interestingly, Zhao and collaborators [20] showed that transcripts encoding LAPs represent one of the most abundant coding sequences of the *Lychas mucronatus* venom, reaching up to 17% of the total toxin-like protein content. This suggests an important role in the venom, such as lipolysis agents or targeting Na^+^ channel currents.

Another coincident Old World venom peptide found in *A. platnicki* was a putative toxin acting as a K^+^-modulating peptide, which could also affect Ca^+2^-releasing nuclear receptors [32]. This small peptide is also found in several scorpion venoms, including the genera *Mesobuthus*, *Buthus*, and *Androctonus*. Other K^+^ channel putative toxins, similar to those from Old World and New World species, were also identified in our analysis [46].

Regarding protein composition, members of the CAP-domain superfamily are commonly found in scorpion venoms [22], and they are the most abundant proteins in some species [47]. Some members of this family of proteins are allergenic [23,24], but in scorpions, their function is still unknown [37]. We found at least two putative CAP-domain proteins in *A. platnicki* venom, one of them with similarity to *Lychas buchari* CAP-Lyc-1, which migrates together with a hyaluronidase in the electrophoresis under native conditions. Interestingly, it has been shown that in the venom of the theraphosid spider *Acanthoscurria natalensis*, a hyaluronidase and a CAP-domain cysteine-rich protein form a complex [35]. The same event has been suggested in insects [48] and scorpions [49]. However, conclusive evidence is required to establish whether this association occurs in this venom and the functionality of that relationship.

The presence of hemocyanins in arthropod venoms has been widely reported [50,51,52]. Although some authors indicate that they are probably hemolymph contaminants [43], there is strong evidence that they could be secreted by venom glands [42,51,53,54,55,56]. We consider it improbable that the venom of *A. platnicki* was contaminated with hemolymph during extraction. We have observed that, in a few cases, electrostimulation causes the outflow of hemolymph between the junctions of the metasoma segments. However, this is an infrequent event. During extraction procedures, we only introduce the sting into the capillary tubes, so the rest of the telson and metasoma remain outside the venom-collecting tube. Therefore, we concluded that the presence of these proteins in *A. platnicki* venom is not a consequence of hemolymph contamination.

Toxic hemocyanin activity against microbial organisms has been widely demonstrated, especially in spiders [34,50], which could indicate that their presence in *A. platnicki* venom might be associated with that function. Contrary to what was observed in the venom gland of the scorpion *Lychas mucronatus* [20,21], in *A. platnicki* venom, we were not able to identify any antimicrobial peptides, nor other non-disulfide-containing peptides for that matter.

Regarding the possibility of hemocyanin fragmentation that could explain their identification at different molecular weights, several scorpion venom proteinases could be associated with post-translational toxin processing [57], and this venom expresses several putative proteolytic enzymes without known function. We identified putative serine, aspartic proteases, and metalloproteinases (Table 1). Likewise, *Lychas mucronatus* venom was shown to express serine and metalloproteinases [20,21], but no specific function was attributed to them.

One of these enzymes found in *A. platnicki* venom was an angiotensin-converting enzyme-like, similar to the *T. serrulatus* ACE-like protein [36]. We confirmed its activity by RP-HPLC using human angiotensin I as a substrate. The appearance of other peptides than the one corresponding to angiotensin II suggests that other venom proteases are also at work. In the case of *T. serrulatus*, seven peptides were produced when angiotensin I was incubated with the venom, but only angiotensin II was formed when, instead of the venom, the purified ACE-like enzyme was incubated with the substrate [36]. This indicates the further action of other venom proteases on the substrate and/or the products. Among the peptides obtained after *T. serrulatus* venom incubation, there were Ang(1–4), Ang(5–10), Ang(1–7), Ang(8–9), Ang(2–8) (also named angiotensin III), and Ang(6–10) [36]. Interestingly, it has been observed that in the human renin–angiotensin system, angiotensin III is produced from angiotensin II by a Zn^+2^-metalloproteinase, and there is another enzyme that acts on both angiotensins I and II, producing Ang(1–7) and Ang(1–9). Ang(1–4), one of the peptides formed by the action of *T. serrulatus* venom, in the case of the human system, is produced from Ang(1–7) by a neprilysin [58]. Then, other peptidases present in the venom of *A. platnicki*, such as aminopeptidases and neprilysins, could be directly cleaving angiotensin I or the peptides released from angiotensin I by the previous action of the ACE-like enzyme.

The biological function of this ACE-like enzyme in scorpion venoms is yet to be fully understood. However, it could play a crucial role in affecting vertebrate predators’ blood pressure or inducing insect neuropeptide processing [36,59]. Furthermore, it has been demonstrated that human angiotensin II could be involved in inflammation and platelet activation by promoting cytokine production by monocytes and macrophages [58].

Another intriguing putative proteolytic enzyme present in this venom was the ubiquitous serine peptidase S1, which has been found in vertebrate and invertebrate venoms with functions associated with fibrinogen, plasminogen, and kininogen cleavage [23]. This peptidase and a putative carboxypeptidase M14, a Zn^+2^-metalloproteinase that plays a role in blood coagulation in organisms such as jellyfish [60], for instance, are good candidates for the in vitro fibrinogenolytic activity displayed by *A. platnicki* venom. However, in our study, we could only confirm a role for metalloproteinases, since EDTA was able to inhibit venom-induced fibrinogenolytic activity. This intricate process highlights the complexity of venom proteases and their potential applications in various biological systems.

In terms of envenoming, there is no further evidence of the effect that *A. platnicki* venom may have on humans. Only one medical case of a sting in Panama [61] is available, where the authors report that the envenoming is mildly toxic to humans but causes intense pain and swelling at the sting site. This observation, the biochemical composition of toxins unraveled here, and the small amount of venom that must be inoculated during the sting suggest that envenoming by this species would not represent a significant medical threat.

In this study, we showed for the first time the general venom composition of a scorpion from the genus *Ananteris*. This characterization is relevant because it has been postulated that members of the *Ananteris* group could be part of a basal clade linking the Old World and the New World Buthidae species, and venom characters could help in the understanding of these scorpions’ phylogenetic relationships. From our venomics analysis, *A. platnicki* venom displays the classical buthid protein family profile, with resemblance to toxins found in Old World genera such as *Lychas*, *Mesobuthus*, and *Isometrus*, in agreement with the molecular-supported phylogenetic affinities between *Ananteris* and these genera [11]. Still, there is evidence of similarity with other venom components of New World buthids, such as *Tityus* and *Centruroides*. This finding suggests that the genus *Ananteris* could represent an ancestral lineage that shared a common ancestor with other buthid scorpions from the New World.

## 4. Materials and Methods

### 4.1. Scorpion Specimens and Venom

Thirty-seven specimens of adult *Ananteris platnicki* (Figure 1) from Hacienda Barú, Puntarenas Province (9°16′20.50″ N, 83°52′46.19″ W), Costa Rica, were manually collected at night. They were transported to the *Laboratorio para la Investigación de Animales Peligrosos* (*LIAP*) facilities (Instituto Clodomiro Picado, Universidad de Costa Rica) and kept in plastic boxes (individually), with insects and insect larvae as food and ad libitum supplement of water.

Venom was collected in capillary tubes after extraction by using telson-electrostimulation. The extraction procedure consisted of placing the telson between two electrodes and applying a discharge of 40–50 V (0.7–0.8 A). A pool of venom samples was collected into a plastic tube, which was lyophilized and stored at −70 °C. The study and animal procedures were approved by the Biodiversity Commission of Universidad de Costa Rica (No. 432-2024).

### 4.2. Electrophoresis and Determination of Enzymatic Activities

Venom was analyzed by 15% SDS-PAGE under reducing conditions (5% 2-mercaptoethanol) in a Mini-Protean system (Bio-Rad, Hercules, CA, USA) at 150 V. To concentrate venom proteins as a single band for some of the mass spectrometry analyses, SDS-PAGE was stopped as soon as the migration front entered into the gel. For native conditions-PAGE, we used commercial precast gels (BioRad, CA, USA, Mini-Protean, catalog no. 4561093), under the manufacturer’s indications. Staining was carried out with Coomassie Blue R-250.

Hyaluronidase activity–zymography was determined as described by Cevallos and collaborators [62] using a 12% SDS-PAGE gel containing 0.5 mg/mL of rooster comb hyaluronic acid (Sigma Chemical Co., St. Louis, MO, USA), incubated in buffer (0.1 M NaCl, 0.1 M sodium phosphate), pH 6.6. Gels were stained with Alcian Blue 8GX (Sigma Chemical Co.).

Zymography to determine proteolytic activity on gelatin was carried out under unreduced conditions SDS-PAGE on 12% gels containing 0.25 mg/mL of type A gelatin (Sigma Chemical Co.). Gels were washed with 1% Triton X-100 for one hour and incubated for 16 hr at 37 °C in 50 mM of Tris–HCl buffer, 5 mM of CaCl_2_, pH 8.0. After incubation, gels were stained with Coomassie Blue R-250.

The venom fibrinogenolytic activity was evaluated by incubating 20 µg of human fibrinogen (Sigma Chemical Co.) with the venom (3 µg) in a final 40 µL volume for 6 h at 37 °C. The experiment was also carried out in the presence of 10 mM of EDTA. Then, the mixture was analyzed by SDS-PAGE (12%) as described, and the degradation of fibrinogen chains was determined by Coomassie blue R-250 staining of the gels [63].

ACE-like activity was determined by RP-HPLC using human angiotensin I (Sigma, A9650) as a substrate and human angiotensin II (Sigma, A9525) as a standard. Accordingly, 20 µmol of the substrate was incubated for 3 hr at 37 °C with 10 µg of venom in PBS (in the absence and presence of 10 mM of EDTA). The sample (200 µL) was applied to a Phenomenex Luna Omega C_18_ column (50 × 2.1 mm, 5 µm particle size) with a C_18_ security guard cartridge (4 × 2 mm) equilibrated with 10% acetonitrile in 0.1% TFA-distilled water, using an Agilent 1220 chromatograph with monitoring at 215 nm. A linear acetonitrile gradient was applied from 10 to 32% to determine the formation of angiotensin II, according to Araújo-Tenorio and collaborators [64]. RP-HPLC fractions collected after the incubation of *A. platnicki* venom with Angiotensin I in the presence or absence of 10 mM of EDTA were dried, redissolved in 50% acetonitrile and 0.1% formic acid, and analyzed by direct infusion (flow rate 5 µL/min) using a Q-Exactive Plus^®®^ mass spectrometer (Thermo Fisher, Santa Clara, CA, USA) with a heated electrospray ionization (HESI) ion source. MS spectra were acquired in positive mode, using 3.9 kV spray voltage, a full MS scan range from 200 to 2500 m/z or 250 to 2500 m/z, 140,000 resolution, and an AGC target of 3 × 10^6^. MS/MS spectra were acquired using a 3.9 kV spray voltage, 140,000 resolution, an AGC target of 3 × 10^6^, and different collision energies and scan ranges, according to each peptide. Multiply charged peptides were fragmented and sequenced manually with the help of PEAKS X^®®^ (Bioinformatics Solutions Inc., Waterloo, ON, Canada).

### 4.3. RP-HPLC for Venom Protein Separation

Two milligrams of venom dissolved in 200 µL of 0.1% TFA (solution A) and centrifuged for 5 min at 10,000 g was separated by reverse-phase HPLC on a 250 × 4.6 mm, 5 µm particle size C_18_ column (Luna Omega, Phenomenex, CA, US) in an Agilent 1220 chromatograph (215 nm). Elution was carried out at 1 mL/min with a linear gradient towards 60% TFA-containing acetonitrile (solution B) for 60 min.

### 4.4. Mass Spectrometry

The crude venom was first analyzed using a ‘shotgun’ MS approach. A sample of 15 μg was dissolved in 25 mM of NH_4_HCO_3_, reduced in solution with 10 mM of dithiothreitol for 30 min at 56 °C, and alkylated with 50 mM of iodoacetamide for 20 min in the dark. Digestion was carried out with sequencing-grade trypsin overnight at 37 °C, in a total volume of 40 μL, and stopped with 0.4 μL of formic acid. A second venom sample was treated similarly but digested instead with V8 protease (G-Biosciences, St. Louis, MO, USA). In addition, some Coomassie-stained venom protein bands obtained from either SDS-PAGE, native conditions-PAGE, or HPLC fractions subjected to SDS-PAGE were in-gel reduced, alkylated, and digested with trypsin as described above, in an automated workstation (Intavis, Tübingen, Germany). All resulting proteolytic peptides were analyzed by nESI-MS/MS using a nano-Easy^®®^ 1200 chromatograph in line with a Q-Exactive Plus^®®^ mass spectrometer (Thermo Fisher, CA, USA). In addition, 5 µL of each digest was loaded on a C_18_ trap column (75 μm × 2 cm, 3 μm particle; PepMap, Thermo), washed with 0.1% formic acid (solution A), and separated at 200 nL/min with a 3 µm particle, 15 cm × 75 µm C_18_ Easy-spray^®®^ analytical column using the following gradient toward solution B (80% acetonitrile, 0.1% formic acid): 1–5% B in 1 min, 5–25% B in 30 min, 25–79% B in 6 min, 79–99% B in 2 min, and 99% B in 6 min, for a total time of 45 min [65]. MS spectra were acquired in positive mode at 1.9 kV, with a capillary temperature of 200 °C, using 1 scan at 400–1600 m/z, a maximum injection time of 100 msec, AGC target of 3 × 10^6^, and orbitrap resolution of 70,000. The top 10 ions with 2–5 positive charges were fragmented with an AGC target of 1 × 10^5^, maximum injection time of 110 msec, resolution of 17,500, loop count of 10, isolation window of 1.4 m/z, and a dynamic exclusion time of 5 s. MS/MS spectra were processed for the assignment of peptide matches to known protein families by similarity with sequences contained in the UniProt/SwissProt database (Scorpions, 2023) using Peaks X^®®^ (Bioinformatics Solutions). Parent and fragment mass error tolerances were set at 15.0 ppm and 0.5 Da, respectively. The carbamidomethylation of cysteine was set as a fixed modification, while the deamidation of glutamine or asparagine, and the oxidation of methionine, were set as variable modifications, allowing up to 3 missed proteolytic cleavages. The filtration parameters for match acceptance were set to FDR < 1%, detection of at least one unique peptide, and −10lgP protein score ≥ 30. The primary raw spectral data were deposited in the PRIDE/Proteome Exchange repository.

## Figures and Tables

**Table 1:** Identified *Ananteris platnicki* venom peptides and proteins assigned to Scorpiones Uniprot database families by MS matching of components obtained with a ‘shotgun’ approach of the crude venom. The detailed information is presented in Supplementary Table S1.

#	Matching Protein, Species	Protein Family	Accession
1	Toxin, *Mesobuthus eupeus*	K^+^ channel/Ca^+2^ channel	A0A5P8U2N2
2	Toxin, *Mesobuthus eupeus*	K^+^ channel	A0A5P8U2Q6
3	Toxin, *Tityus obscurus*	K^+^ channel	A0A1E1WVV4
4	Neurotoxin LmNaTx9, *Lychas mucronatus*	Na^+^ channel	A0A0U1S5U0
5	Neurotoxin LmNaTx12, *Lychas mucronatus*	Na^+^ channel	A0A0U1SJ71
6	Neurotoxin LmNaTx18, *Lychas mucronatus*	Na^+^ channel	A0A0U1SN99
7	LCN-type CS-alpha/beta domain-containing protein, *Isometrus maculatus*/LmNaTx19, *Lychas mucronatus*	Na^+^ channel, LAPβ	A0A0U1S617
8	LCN-type CS-alpha/beta domain-containing protein, *Isometrus maculatus/* LmNaTx25 *Lychas mucronatus*	Na^+^ channel, LAPα	A0A0U1SPD0
9	LCN-type CS-alpha/beta domain-containing protein, *Isometrus maculatus/*LmNaTx30, *Lychas mucronatus*	Na^+^ channel	A0A0U1TZ19
10	LCN-type CS-alpha/beta domain-containing protein, *Isometrus maculatus*	Na^+^ channel	A0A0U1S617
11	Venom peptide meuPep27, *Mesobuthus eupeus*	Na^+^ channel, LAPα	A0A146CJ25
12	Putative Na+ channel toxin, *Superstitionia donensis*	Na^+^ channel	A0A1V1WBQ9
13	Sodium channel toxin Ts1, *Tityus serrulatus*	Na^+^ channel	A0A7S8RFZ4
14	Neurotoxin, *Tityus obscurus*	Na^+^ channel	A0A1E1WW03
15	Alpha-amylase, *Tityus obscurus*	Amylase	A0A1E1WVL9
16	Alpha-amylase, *Hadrurus spadix*	Amylase	A0A1W7RB82
17	Cysteine-rich secretory protein, *Centruroides hentzi*	CRISP	A0A2I9LNV7
18	CAP-Lyc-1, *Lychas buchari*	CRISP	T1DPC1
19	Hyaluronidase, *Androctonus crassicauda*	Hyaluronidase	A0A7T9L322
20	Glyceraldehyde-3-phosphate dehydrogenase, *Hadrurus spadix*	Oxidorreductase	A0A1W7RAH3
21	Putative peptidyl-glycine alpha-hydroxylating monooxygenase, *Tityus serrulatus*	Oxidase	A0A7S8RGB6
22	Angiotensin-converting enzyme, *Tityus serrulatus*	Peptidase	A0A1E1WWB8
23	Neprilysn, *Hadrurus spadix*	Peptidase	A0A1W7RA38
24	Metalloproteinase, *Centruroides hentzi*	Metalloproteinase	A0A2I9LP89
25	Metalloproteinase, *Centruroides hentzi*	Metalloproteinase	A0A2I9LP68
26	Metalloproteinase, *Tityus obscurus*	Metalloproteinase	A0A1E1WVW3
27	Peptidase M14, *Isometrus maculatus*	Carboxypeptidase	A0A0U1SF04
28	Cathepsin spartic protease, *Centruroides hentzi*	Aspartic protease	A0A2I9LNV0
29	Peptidase S1 domain-containing protein, *Isometrus maculatus*	Serine protease	A0A0U1S633
30	Aminopeptidase, *Hadrurus spadix*	Aminopeptidase	A0A1W7RAL3
31	Phospholipase A2 *Tityus obscurus*	Phospholipase A_2_	A0A1E1WVV6
32	Phospholipase A2, *Hadrurus spadix*	Phospholipase A_2_	A0A1W7RA16
33	Venom protein, *Centruroides hentzi/Isometrus maculatus*	unclassified	A0A2I9LPX8
34	Venom protein, *Isometrus maculatus*	unclassified	A0A0U1S614
35	Venom protein, *Mesobuthus eupeus*	unclassified	E4VP36
36	Ectonucleotide pyrophosphatase/phosphodiesterase, *Tityus obscurus*	Phosphodiesterase	A0A1E1WVL0
37	Hemocyanins, *Tityus obscurus/Hadrurus spadix/Pandinus imperator*	Hemocyanin	A0A1E1WWC5

## Data Availability

The raw data supporting the conclusions of this article will be made available by the authors upon request.

## Supplementary Materials

The following supporting information can be downloaded at: https://www.mdpi.com/article/10.3390/toxins16080327/s1, Table S1. Proteomic profiling of the venom of *Ananteris platnicki* (Costa Rica).
